# Application of an LC–MS/MS Method for the Simultaneous Quantification of Homovanillic Acid and Vanillylmandelic Acid for the Diagnosis and Follow-Up of Neuroblastoma in 357 Patients

**DOI:** 10.3390/molecules26113470

**Published:** 2021-06-07

**Authors:** Narae Hwang, Eunbin Chong, Hyeonju Oh, Hee Won Cho, Ji Won Lee, Ki Woong Sung, Soo-Youn Lee

**Affiliations:** 1 Department of Laboratory Medicine and Genetics, Samsung Medical Center, Sungkyunkwan University School of Medicine, Seoul 06351, Korea; Narae12@gmail.com (N.H.); eunbin.chong@samsung.com (E.C.); hyeonju.oh@samsung.com (H.O.); 2 Department of Pediatrics, Samsung Medical Center, Sungkyunkwan University School of Medicine, Seoul 06351, Korea; hw87.cho@samsung.com (H.W.C.); leejw.lee@samsung.com (J.W.L.); 3 Department of Clinical Pharmacology & Therapeutics, Samsung Medical Center, Sungkyunkwan University School of Medicine, Seoul 06351, Korea; 4 Department of Health Science and Technology, Samsung Advanced Institute of Health Science and Technology, Sungkyunkwan University, Seoul 06351, Korea

**Keywords:** mass spectrometry, validation, vanillylmandelic acid, homovanillic acid, neuroblastoma, urine

## Abstract

Homovanillic acid (HVA) and vanillylmandelic acid (VMA) are end-stage metabolites of catecholamine and are clinical biomarkers for the diagnosis of neuroblastoma. For the first time in Korea, we implemented and validated a liquid chromatography tandem mass spectrometry (LC–MS/MS) assay to measure urinary concentrations of HVA and VMA according to Clinical and Laboratory Standards Institute guidelines. Our LC–MS/MS assay with minimal sample preparation was validated for linearity, lower limit of detection (LOD), lower limit of quantification (LLOQ), precision, accuracy, extraction recovery, carryover, matrix effect, and method comparison. A total of 1209 measurements was performed to measure HVA and VMA in spot urine between October 2019 and September 2020. The relationship between the two urinary markers, HVA and VMA, was analyzed and exhibited high agreement (89.1% agreement, kappa’s k = 0.6) and a strong correlation (Pearson’s *r* = 0.73). To our knowledge, this is the first study to utilize LC–MS/MS for simultaneous quantitation of spot urinary HVA and VMA and analyze the clinical application of both markers on a large scale for neuroblastoma patients.

## 1. Introduction

Neuroblastoma is the most common extracranial solid tumor in children and accounts for 7–10% of pediatric malignancies [1]. Neuroblastoma cells synthesize excess catecholamines including norepinephrine, epinephrine, and dopamine [2]. These cells contain small amounts of storage vesicles; hence, storage of synthesized catecholamines is limited [2]. Therefore, most catecholamines produced by neuroblastoma cells are metabolized to 4-hydroxy-3-methoxyphenylglycol (MHPG) by catechol-*O*-methyltransferase (COMT) and monoamine oxidase (MAO) [3]. MHPG secreted by neuroblastoma cells is metabolized to vanillylmandelic acid (VMA) mainly by hepatic alcohol dehydrogenase [3]. Dopamine is completely metabolized to homovanillic acid (HVA) by COMT and MAO within neuroblastoma cells [4]. These metabolites of catecholamines are produced in relatively large amounts with micromolar concentrations and are mainly excreted in urine, allowing ease of measurement [4]. HVA and VMA serve as useful clinical biomarkers for diagnosis and follow-up of neuroblastoma [5]. Biochemical detection of urinary HVA and VMA allows diagnosis of neuroblastoma with high sensitivity (66–100%) and specificity (>99%) when the two markers are combined [6,7]. High-performance liquid chromatography coupled with electrochemical detection (HPLC–ECD) has been utilized for quantification of HVA and VMA in urine. The HPLC–ECD method requires a complex sample extraction such as solid-phase or liquid–liquid extraction and has a long data acquisition time (approximately 10 to 20 min) [8]. Recently, an LC–MS/MS method was introduced for the quantification of HVA and VMA. This method allows simple sample preparation procedures, such as the dilute-and-shoot method, and rapid data acquisition (3 to 8 min), which are useful features in a clinical environment, especially when a large number of tests have to be performed. [8,9,10,11]. Up to now, HPLC–ECD has been predominantly used in Korea to quantify urinary HVA and VMA. Two laboratories utilize LC–MS/MS to measure urinary VMA, and only our laboratory measures both analytes simultaneously [12]. Since HVA and VMA are both used as biomarkers of neuroblastoma and have similar characteristics, it is more convenient to quantitate both analytes in the same specimen [13]. Clinically, the utility of HVA and VMA quantitation in spot urine is increasing due to the convenience of sample collection. However, to the best of our knowledge, there are no studies regarding the clinical application of urinary HVA and VMA in neuroblastoma patients on a large scale.

Herein, we validated an LC–MS/MS method for simultaneous measurement of urinary HVA and VMA for diagnosis and follow-up of neuroblastoma patients and analyzed the clinical applications of HVA and VMA using 1209 measurements.

## 2. Results and Discussion

### 2.1. Method Validation

The total chromatographic run time was 6 min/sample. The calibration curves were linear over 0.5–100 mg/L, with a coefficient of linearity (*R^2^*) >0.99 for both HVA and VMA (Table 1). The HVA and VMA LLOQ and LOD were 0.5 mg/L and 0.1 mg/L, respectively. Intra-assay CVs (*n* = 5) were 3.7 and 2.5% for HVA and 1.8 and 1.1% for VMA, respectively; inter-assay CVs (*n* = 25) were 3.9 and 3.6% for HVA and 4.1 and 3.0% for VMA at low and high concentrations, respectively (Table 1). The method showed good accuracy with a bias of −9.1% to 11.3% for HVA and −9.9% to 6.3% for VMA. Extraction recoveries ranged from 97.0% to 107.0% for HVA and 97.0% to 106.0% for VMA. Although an ion suppression effect was observed, the matrix effect bias (%) with IS normalization was acceptable with values from 93.0% to 112.0% for HVA and 97.0% to 110.0% for VMA. No significant carryover effect was observed. Our laboratory previously used HPLC–ECD for measurement of VMA for more than 10 years. Therefore, we performed method comparison between HPLC–ECD and LC–MS/MS and obtained comparable results for urinary concentrations of VMA in 41 urine specimens (*r* = 0.98, *p* < 0.0001, Figure S1, Supplementary Materials).

There have been several attempts to measure urinary HVA and VMA by LC–MS/MS [8,9,10,11]. Most studies used a C18 or C16 column with an acetonitrile or methanol gradient (Table 1 and Table S1, Supplementary Materials). Our method development for measurement of HVA and VMA was comparable to those of previous studies in terms of AMR, precision, accuracy, LLOQ, run time, and sample volume. Two studies evaluated matrix effect with post-column infusion and reported no matrix effect in their study [8,9]. Our study used a post-extraction spiking method to evaluate the matrix effect, which used IS to compensate for the possible ion suppression. Recovery was comparable to that in other studies [9,10].

More than 1000 urine specimens are submitted annually to our laboratory for HVA and VMA quantitation; therefore, we validated a dilute-and-shoot method with a rapid turnaround time and simpler preparation procedures. We analyze about 70 to 80 urine specimens per batch for quantitation of HVA and VMA weekly, and sample preparation time takes about 30 min. Since total analysis time takes about 7–8 h per batch, sample analysis by LC–MS/MS is usually performed overnight.

The strength of this study is that we performed 1209 HVA and VMA measurements in neuroblastoma patients on a large scale with good analytical performance.

### 2.2. Clinical Application

**Figure 1:**
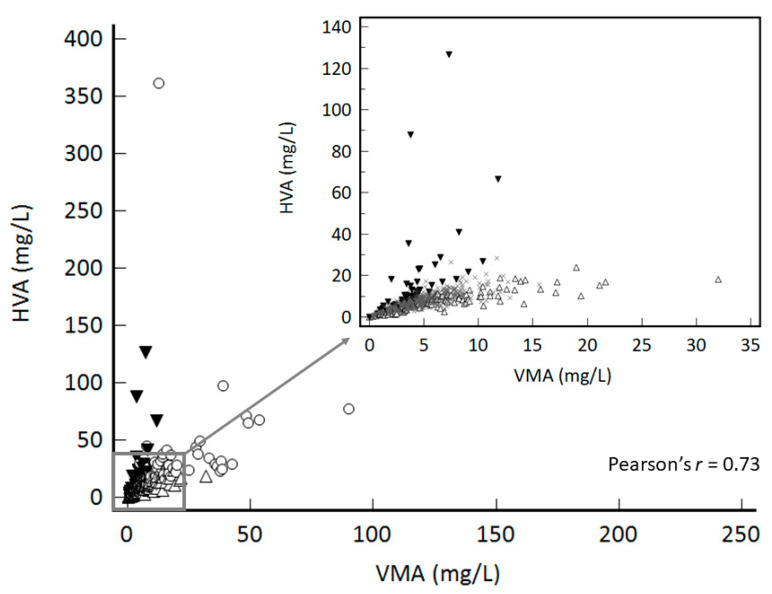
Relationship between 1209 VMA and HVA concentrations in 357 patients.

The scatter plot of 1209 measurements of HVA and VMA in spot urine of 357 neuroblastoma patients over 13 months is presented in Figure 1. The numbers of patients who repeated measurement more than twice and more than five times during the period were 265 (74.2%) and 85 (23.9%) patients, respectively, indicating that HVA and VMA measurements in spot urine are routinely used for monitoring of patients with neuroblastoma.

In this study, disease monitoring was performed by measuring HVA and VMA in spot urine specimens. Although the standard urine sampling for HVA and VMA measurement is a 24 h urine specimen, sample collection is troublesome and invasive for pediatric patients since catheterization is often required. HVA and VMA concentrations in spot urine, expressed as the ratio of metabolite to creatinine concentrations, have a good correlation with concentrations in 24 h urine specimen [14]. Age-specific reference ranges for HVA and VMA are normalized by urine creatinine concentration [4]. Therefore, spot urine specimens are more appropriate for pediatric patients for measurement of HVA and VMA [14].

Comparison of HVA and VMA in spot urine exhibited moderate agreement (89.1% agreement, kappa’s k = 0.6) and a strong correlation (Pearson’s *r* = 0.73) (Figure 1).

We analyzed 1209 HVA and VMA measurements in spot urine specimens by applying age-specific reference ranges (Table 2) [4]. Of these, 1077 (89%) showed accordant results for HVA and VMA, while 132 (11%) results showed discrepant results. To compare the values among groups, the indices of analytes were obtained by dividing HVA and VMA results with the upper limit of age-specific reference ranges. When the HVA and VMA results were both positive, the HVA and VMA indices were well above the age-specific reference ranges. In contrast, the results were borderline with respect to the upper limit of age-specific reference ranges when HVA and VMA results showed discrepancies.

Until now, few studies have evaluated the sensitivity and specificity of HVA and VMA in neuroblastoma patients at disease onset, but there are no studies regarding utility of HVA and VMA quantitation in spot urine for monitoring of neuroblastoma patients [7,15,16].

Among 1209 measurements, 20 were performed for patients at onset of neuroblastoma, and diagnosis was confirmed by histopathology and/or imaging techniques (Table 3). The other 1189 measurements were performed for monitoring of neuroblastoma patients at follow-up. Median age of 20 neuroblastoma patients at onset of disease was 2 (range, 1 month–12 years).

Concentration of urine creatinine showed severe variation in the range of 5.1 mg/dL to 121.3 mg/dL; thus, normalization of HVA and VMA results with concentration of urine creatinine was inevitable. Volume of 24 h urine also showed variation with range of 48 mL to 1600 mL, which implies that the collection procedure might not be accurate, affecting the validity of HVA and VMA results in 24 h urine.

Among 20 neuroblastoma patients, 14 tested positive both for HVA and VMA, two tested negative both for HVA and VMA, and four showed discrepant results between HVA and VMA (Table 2). Among 14 patients with positive HVA and VMA results, 11 (79%) were stage 4 (patients 4–14), while three patients were stage 2 (patients 1–3). This indicates that HVA and VMA results tend to be more accordant in high disease stages. Two patients (patients 15 and 16) tested negative for both HVA and VMA, and their histopathology was confirmed as ganglioneuroblastoma. Ganglioneuroblastoma is considered a favorable risk group in the international neuroblastoma risk group [17]. Moreover, these two patients both had zero metaiodobenzylguanidine (MIBG) score, which implies that the two false-negative cases could be non-secreting type [18]. MIBG is a catecholamine analog that binds to iodine-123, and catecholamine-producing tumors like neuroblastoma are known to avidly take up this radiopharmaceutical drug, which is monitored by scintigraphy [17].

Among the four patients with discrepant results, three patients tested positive for HVA and negative for VMA. These patients were all stage 4 patients with MYCN amplification, and the VMA in 24 h urine was negative (patients 17–19). It is interesting that all of them had mild (MIBG) involvement, which implies that VMA production by neuroblastoma is low in these patients [17]. One patient, who tested negative for HVA and positive for VMA, was stage 2 (patient 20). Previous studies reported that the sensitivity of HVA is low compared to that of VMA in stage 1 and 2 neuroblastoma [15,16].

Three (patients 8, 15, and 16) of 20 newly diagnosed patients were ganglioneuroblastoma patients. Although ganglioneuroblastoma is a less malignant tumor, it can present as advanced cancer when patients have poor prognostic factors, as in patient 8 in our case. Patient 8 was stage 4 ganglioneuroblastoma and tested positive for HVA and VMA; the patient had a 17q gain on fluorescence in situ hybridization (FISH) analysis, which is a poor prognostic factor.

## 3. Materials and Methods

### 3.1. Materials

HVA and VMA standards were purchased from Sigma Aldrich (St. Louis, MO, USA). LC–MS/MS-grade acetonitrile, methanol, and distilled water were purchased from Burdick & Jackson (Muskegon, MI, USA). Formic acid and hydrochloric acid were purchased from Sigma-Aldrich (St. Louis, MO, USA). Mass Spect Gold Human Urine (MSG5000) was purchased from Golden West Diagnostics (Temecula, CA, USA). HVA-d_5_ and VMA-d_3_ were purchased from CDN Isotopes (Pointe-Clarie, Quebec, QC, Canada). Lyphocheck Urine Quality control (QC) samples were obtained from Bio Rad (Hercules, CA, USA).

### 3.2. Preparation of Calibrators, Quality Controls, and Patient Samples

Stock solutions (1 mg/mL) were prepared by dissolving HVA and VMA in 70% (*v*/*v*) methanol; these were frozen and stored at −80 °C until use. The stock solutions were diluted with blank urine (Mass Spect Gold Human Urine) to prepare the calibrators at six concentrations (0.5, 1.0, 5.0, 10, 50, and 100 mg/L HVA or VMA). Stock solutions of internal standards (IS) (2 mg/mL) were prepared by dissolving HVA-d_5_ and VMA-d_3_ in methanol. Quality control (QC) samples were prepared at two concentrations (1.7 mg/L and 15.7 mg/L for HVA; 2.7 mg/L and 15.0 mg/L for VMA) with 0.05% hydrochloric acid as per the manufacturer’s instructions.

For the LC–MS/MS assay, 50 μL of patient urine samples or blank/calibrators/controls and 100 μL of protein precipitation solution at −20 °C (50% acetonitrile in methanol) including IS (5 μg/mL) were added to Eppendorf tubes. The tubes were sealed and vortexed for 30 s and then centrifuged at 20,600× *g* for 10 min. A volume of 20 μL of the supernatant was transferred to the wells of a 2.0 mL polypropylene 96-deep-well plate. Next, 100 μL of distilled water was added to each well of the plate, mixed for 30 s, and then transferred into an autosampler at an injection volume of 10 μL.

### 3.3. Instrument Conditions

**Figure 2:**
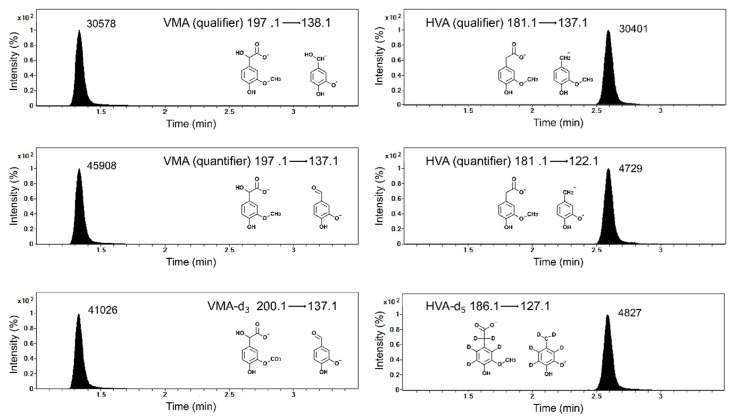
Representative LC–MS/MS chromatograms of blank urine spiked with HVA (10 μg/mL), VMA (10 μg/mL), and their internal standards (HVA-d_5_ and VMA-d_3_, 5 μg/mL).

Chromatographic separation was carried out using an Agilent 1260 HPLC system (Agilent Technologies, Santa Clara, CA, USA) coupled to an Agilent 6460 tandem mass spectrometer equipped with a polar embedded C18 with trimethylsilane endcapping LC column (2.0 × 100 mm, 2.5 μm; Phenomenex, Torrance, CA, USA). Mobile phases consisted of water containing 0.1% formic acid (mobile A) and acetonitrile containing 0.1% formic acid (mobile B). The flow rate was 300 μL/min. Gradient elution is summarized in Table 4. Quantitative analysis was performed in multiple reaction monitoring (MRM) mode with a jet stream electrospray ionization source operating in negative ion mode. MRM transitions of HVA, HVA-IS, VMA, and VMA-IS are provided in Figure 2. Optimized MS instrument settings were as follows: source temperature, 325 °C; capillary voltage, 3.5 kV; cone gas flow, 11 L/min; collision energy, 25 V; fragmentor energy, 107 V; dwell time, 50 ms. Quantitation was performed using the peak area ratio of HVA and VMA to their IS using MassHunter Workstation software (version B.06, Agilent Technologies, Santa Clara, CA, USA).

### 3.4. Method Validation

We validated an LC–MS/MS assay for simultaneous measurement of HVA and VMA according to CLSI guidelines [8,23,24]. Calibration linearity was evaluated by least-squares linear regression using six concentrations of HVA and VMA (0.5, 1, 5, 10, 50, and 100 μg/mL) standards in synthetic urine. The limit of detection (LOD) was determined as the lowest concentration assayed with a signal-to-noise ratio (SNR) >3. To determine the lower limit of quantification (LLOQ), HVA and VMA standards were spiked into blank urine and tested in 10 replicates. The LLOQ was determined with a signal-to-noise ratio >10; the coefficient of variation (CV) was <20%, and bias was <20% in replicate assays [23,24]. Intra-assay precision was assessed using five replicates of two QC samples for HVA and VMA (1.7 and 15.7 mg/L for HVA; 2.7 mg/L and 15.0 mg/L for VMA). Inter-assay precision was determined over 25 days (Table 1). The matrix effect and extraction recovery were evaluated by post-extraction spike method with 10 urine samples of neuroblastoma patients at two concentrations of HVA and VMA (5, 50 μg/mL) and IS (HVA-d_5_ and VMA-d_3_, 5 μg/mL). Because the urine samples contained endogenous VMA and HVA, the peak areas of the analytes needed to be adjusted by subtracting the endogenous peak area from the peak area of the analytes. Then, adjusted peak area was divided by the area of pure analyte in distilled water to calculate the matrix effect. The matrix effect was observed due to ionization suppression. After normalizing the peak area to the area of IS, matrix effects were considered acceptable as matrix effect bias (%) with IS normalization was within ±15%. Extraction recovery was calculated as the ratio of the peak areas before and after spiking. Carryover was evaluated with a blank sample after running the highest concentration calibrator containing 100 mg/L of HVA and VMA. The responses of analytes in blank samples were <20% in comparison with those in the LLOQ sample. Accuracy was evaluated with proficiency testing materials (13 College of American Pathologists (CAP) and six Korean Association of External Quality Assessment Service (KEQAS) materials). For method comparison, VMA concentrations in spot urine were measured using the Agilent 1200 HPLC system (Agilent Technologies, Santa Clara, CA, USA) coupled to a Coulochem^®^ III ECD (ESA, Chelmsford, MA, USA). The data were analyzed using MedCalc Statistical Software version 19.0.3 (MedCalc Software bvba, Ostend, Belgium).

### 3.5. Clinical Application

From October 2019 to September 2020, 1209 analyses for 357 neuroblastoma patients were performed to quantitate HVA and VMA in spot urine at Samsung Medical Center. HVA and VMA were reported as mg/g creatinine after measuring urine creatinine with the automatic kinetic Jaffe method on the Cobas INTEGRA^®^ 800 (Roche Diagnostics, Mannheim, Germany).

Demographic and clinical information of sex, age, and laboratory test results was obtained by reviewing the patients’ electronic medical records. We analyzed HVA and VMA results in spot urine for neuroblastoma patients at the onset of disease.

## 4. Conclusions

In the present study, our simple and rapid LC–MS/MS method allowed simultaneous quantitation of HVA and VMA in spot urine in large cohort of patients with neuroblastoma. Analysis of clinical data revealed that HVA and VMA measurement in spot urine is a useful biomarker in clinical practice for diagnosis and monitoring of neuroblastoma patients. To our knowledge, this is the first approach to analyze the large-scale clinical application of HVA and VMA in spot urine of neuroblastoma patients using LC–MS/MS.

## Figures and Tables

**Table 1:** Summary of analytical performance of LC–MS/MS methods for quantification of HVA and VMA.

Reference	Item	LOD (mg/L)	LLOQ (mg/L)	AMR (mg/L)	Precision (Intra-Assay) CV%	Precision (Inter-Assay) CV%	Accuracy (Bias %)	Matrix Effect	Extraction Recovery	Clinical Specimen Number ^a^
This study	HVA	0.1	0.5	0.5–100.0	2.5–3.7	3.6–3.9	−9.1–11.3	93–112%	97–107%	1209
	VMA	0.1	0.5	0.5–100.0	1.1–1.8	3.0–4.1	−9.9–6.3	97–110%	97–106%	
Clark et al., 2017 [8]	HVA	0.1	0.5	0.5–100.0	0.8–2.7	1.6–3.8	<15	None *	NA	NA
	VMA	0.2	0.5	0.5–100.0	1.0–2.7	1.9–4.1	<15		NA	
Shen et al., 2019 [9]	HVA	0.3	0.1	0.1–182.2	3.7–3.8	2.3–3.8	<13.3	None *	86–100%	19
	VMA	0.3	0.1	0.1–99.1	1.4–3.9	2.2–2.9	11.4		85–109%	
Grouzmann et al., 2018 [10]	HVA	NA	0.1	0.1–36.4	4.0–8.0	4.1–4.7	1.8	NA	100%	NA
	VMA	NA	0.1	0.1–39.6	2.3–2.6	5.1	−3.8	NA	100%	
Manini et al., 2000 [11]	HVA	0.03	0.1	0.1–25.0	1.4–3.4	2.6–5.2	2.8–3.9	NA	NA	NA
	VMA	0.07	0.5	0.5–50.0	0.8–1.2	1.1–1.8	2.1–3.3	NA	NA	

Abbreviations: AMR, analytical measurement range; CV, coefficient of variation; HVA, homovanillic acid; LOD, lower limit of detection; LLOQ, lower limit of quantitation; NA, not available; VMA, vanillylmandelic acid; * post-column infusion; ^a^ number of samples generated from neuroblastoma patients.

**Table 2:** HVA and VMA measurements in combination for detection and monitoring of neuroblastoma.

Marker Correlation		*N* (%)	HVA, mg/g Cr, Median (Range)	VMA mg/g Cr, Median (Range)	HVA Index, Median * (Range)	VMA Index, Median * (Range)
Accordance	HVA (+) VMA (+)	127 (10.5)	35.1 (9.8–438.0)	20.1 (5.8–324.8)	1.6 (1.0–20.5)	1.9 (1.0–29.0)
	HVA (−) VMA (−)	950 (78.5)	8.4 (<0.5–27.2)	5.2 (<0.5–16.5)	0.5 (0–1.0)	0.6 (0–1.0)
Discrepancy	HVA (+) VMA (−)	43 (3.6)	23.9 (5.5–111.0)	6.4 (2.8–15.3)	1.2 (1.0–4.7)	0.8 (0.4–1.0)
	HVA (−) VMA (+)	89 (7.4)	13.4 (3.5–28.9)	11.7 (4.4–38.1)	0.8 (0.4–1.0)	1.3 (1.0–3.6)
Total		1209	9.5 (<0.5–438.0)	5.7 (<0.5–324.8)	0.6 (0–20.5)	0.7 (0–29.0)

* The HVA and VMA indices were calculated by dividing each result by the upper limit of age-specific reference ranges. Upper limit of age-specific reference ranges of HVA [4]: 0–3 months, <35.0 mg/g Cr; 3–6 months, <32.1 mg/g Cr; 6–12 months, <31.4 mg/g Cr; 1–2 years, <27.3 mg/g Cr; 2–5 years, <23.5 mg/g Cr; 10–15 years, <9.7 mg/g Cr; 15–20 years, <5.8 mg/g Cr; 20–25 years, <5.2 mg/g Cr. Upper limit of age-specific reference ranges of VMA [4]: 0–3 months, <18.9 mg/g Cr; 3–6 months, <21.8 mg/g Cr; 6–12 months, <16.9 mg/g Cr; 1–2 years, <15.5 mg/g Cr; 2–5 years, <10.3 mg/g Cr; 10–15 years, <5.6 mg/g Cr; 15–20 years, <4.3 mg/g Cr; 20–25 years, <4.6 mg/g Cr. Abbreviations: Cr, creatinine; d, days; HVA, homovanillic acid; m, months; VMA, vanillylmandelic acid; SD, standard deviation; y, years.

**Table 3:** Clinical characteristics and laboratory findings of 20 neuroblastoma patients at the onset of disease.

Patient no.	Sex	Age at Dx.	No. of Measurements	Spot Urine				24 h Urine		Urine Cr (mg/dL)	24 h Urine Volume (mL)	Cancer Staging	*MYCN*/1p/11q/17q ^e^	MIBG ^f^
				HVA (mg/L)	HVA (mg/g Cr) ^a^	VMA (mg/L)	VMA (mg/g Cr) ^b^	HVA (mg/Day) ^c^	VMA (mg/Day) ^d^					
1	M	1 m	6	2.7	**33.7**	2.6	**32.5**	1.6	1.8	8.0	559	2	−/−/−/−	1
2	M	8 m	1	7.1	**36.8**	7.0	**36.1**	**3.1**	2.3	19.3	1600	2	−/−/−/−	0
3	F	12 d	9	24.5	**108.2**	38.5	**170.2**	ND	**8.8**	22.6	718	2	−/−/−/+	1
4	M	6 m	14	2.4	**47.1**	1.3	**25.2**	ND	**3.2**	5.1	410	4	−/−/−/−	0
5	F	1 y	11	11.2	**52.7**	7.9	**37.3**	ND	**8.4**	21.2	1189	4	−/−/−/−	1
6	F	4 y	2	27.0	**32.0**	36.4	**43.2**	4.1	**5.5**	84.2	653	4	−/NA	18
7	M	2 y	6	10.4	**70.6**	7.8	**52.9**	**32.9**	**20.1**	14.8	1000	4	−/−/+/−	18
8	M	6 y	3	64.6	**69.8**	49.2	**53.1**	**41.6**	**27.4**	92.6	910	4(G)	−/−/−/+	9
9	F	9 m	13	5.1	**46.9**	7.6	**69.5**	ND	**10.8**	11.0	646	4	−/−/−/+	2
10	M	5 y	5	109.9	**90.6**	161.8	**133.4**	ND	**35.6**	121.3	1055	4	−/−/−/−	10
11	F	8 y	5	218.2	**191.7**	219.9	**193.2**	ND	**72.0**	113.8	1450	4	−/−/+/+	15
12	F	2 y	8	274.3	**377.2**	206.4	**283.9**	**61.1**	**57.0**	72.7	1150	4	−/+/+/−	1
13	F	2 y	11	70.7	**438.0**	48.3	**298.8**	ND	**34.4**	16.2	48	4	−/−/+/−	26
14	M	2 y	2	77.3	**279.4**	89.9	**324.8**	**23.8**	**31.7**	27.7	550	4	−/−/−/−	28
15	F	6 y	9	6.5	11.7	2.6	4.7	ND	0.9	55.9	187	2(G)	−/−/−/−	0
16	F	7 y	1	17.7	15.2	8.3	7.1	ND	ND	116.8	ND	3(G)	−/−/−/−	0
17	M	2 y	4	18.3	**66.6**	2.0	7.2	ND	1.0	27.5	783	4	+/+/−/−	1
18	F	7 y	3	35.5	**74.8**	3.6	7.5	**17.7**	2.4	47.5	888	4	+/+/−/−	1
19	F	1 y	2	23.3	**43.0**	4.6	8.5	**5.6**	1.5	54.1	1093	4	+/−/−/−	2
20	F	1 y	4	6.9	20.7	7.8	**23.4**	ND	**3.8**	33.4	1400	2	−/−/+/−	1

Abbreviations: Cr, creatinine; Dx, diagnosis; F, female; G, ganglioneuroblastoma; M, male; m, months; MIBG, metaiodobenzylguanidine involvement; MYCN, MYCN amplification; NA, not available; no., number; ND, not done; Pt, patient; y, years. *MYCN* amplification is a poor prognosis factor which is involved with rapid diesease progression and poor reponse to therapy [19]. In this cohort, three stage 4 patients (5%) carried *MYCN* amplification. 1p—deletion, 11q—deletion, and 17q—gain are also involved with poor prognosis, and three patients (5%), five patients (25%), and four patients (20%) carried the chromosomal aberation, respectively [20,21]. Positive results of HVA and VMA are bolded. ^a^ Upper limit of age-specific reference ranges of HVA in spot urine [4]: 0–3 months, <35.0 mg/g Cr; 3–6 months, <32.1 mg/g Cr; 6–12 months, <31.4 mg/g Cr; 1–2 years, <27.3 mg/g Cr; 2–5 years, <23.5 mg/g Cr; 10–15 years, <9.7 mg/g Cr; 15–20 years, <5.8 mg/g Cr; 20–25 years, <5.2 mg/g Cr. ^b^ Upper limit of age-specific reference ranges of VMA in spot urine [4]: 0–3 months, <18.9 mg/g Cr; 3–6 months, <21.8 mg/g Cr; 6–12 months, <16.9 mg/g Cr; 1–2 years, <15.5 mg/g Cr; 2–5 years, <10.3 mg/g Cr; 10–15 years, <5.6 mg/g Cr; 15–20 years, <4.3 mg/g Cr; 20–25 years, <4.6 mg/g Cr. ^c^ Upper limit of age-specific reference ranges of HVA in 24 h urine [4]: 0–1 years, <2.8 mg/day; 2–4 years, <4.7 mg/day; 5–9 years, <5.4 mg/day; 10–19 years, <8.7 mg/day; adults, <8.8 mg/day. ^d^ Upper limit of age-specific reference ranges of VMA in 24 h urine [4]: 0–1 years, <2.3 mg/day; 2–4 years, <3.0 mg/day; 5–9 years, <3.5 mg/day; 10–19 years, <6.0 mg/day; adults, <6.8 mg/day. ^e^ *MYCN* amplification, 1p—deletion, 11q—deletion, and 17q—gain results obtained from fluorescence in situ hybridization (FISH) analysis. ^f^ MIBG involvement was scored according to Modified Curie Scoring Method in 10 different sites including nine skeletal sites (head, chest, T-spine, L-spine, pelvis, upper arms, lower arms, femurs, and lower legs) and an additional 10th site for soft-tissue lesions. Lesions were scored as follows: 0, no MIBG involvement; 1, one MIBG-avid lesion present; 2, more than one MIBG-avid lesion present; 3, MIBG avidity in a lesion that occupied >50% of an individual site [22]. Clinical data for neuroblastoma patients with large-scale quantitation of HVA and VMA using LC–MS/MS have rarely been reported. The present study reported 1209 HVA and VMA measurements and analyzed the clinical application of HVA and VMA quantification in spot urine specimens. Since spot urine specimens are more accessible for pediatric neuroblastoma patients than 24 h urine specimens, it is necessary to assess the clinical utility of HVA and VMA quantitation in spot urine.

**Table 4:** Gradient conditions for chromatographic separation for HVA and VMA quantification.

			Mobile Phase	
Time Segment	Time (min)	Flow Rate (mL/min)	%A *	%B ^†^
1	Initial	0.3	85	15
2	0.5		85	15
3	3.0		10	90
4	4.0		10	90
5	4.1		85	15

* 0.1% Formic acid in distilled water; ^†^ B: 0.1% formic acid in acetonitrile. Abbreviation: HVA, homovanillic acid; VMA, vanillylmandelic acid.

## Data Availability

The data presented in this study are available on request from the corresponding author.

## Supplementary Materials

The following are available online at: Figure S1. Comparison plots between LC-MS/MS and HPLC-ECD. Table S1. Summary of LC–MS/MS methods for quantification of HVA and VMA.
