# Supplementary material for: Application of an LC–MS/MS Method for the Simultaneous Quantification of Homovanillic Acid and Vanillylmandelic Acid for the Diagnosis and Follow-Up of Neuroblastoma in 357 Patients
Source: Molecules. 2021 Jun 7;26(11):3470. doi: 10.3390/molecules26113470 (PMC8201085; doi:10.3390/molecules26113470)
Supplement: Supplementary file 1 [file molecules-26-03470-s001.zip › molecules-1196877-supplementary.pdf]

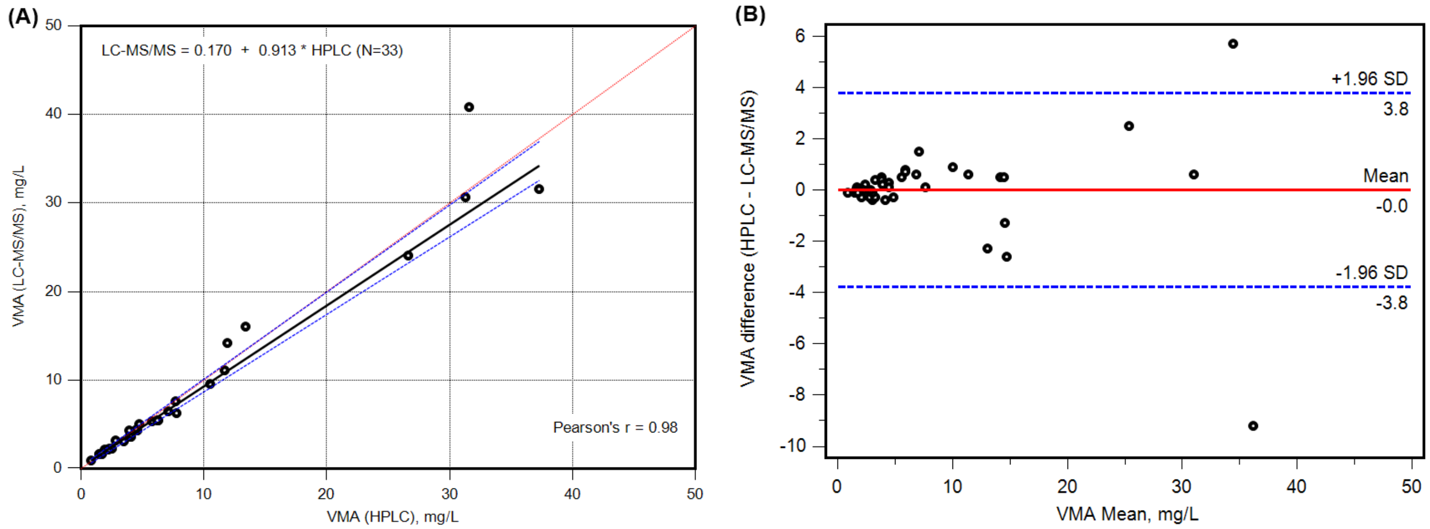

Supplemental Figure 1. Comparison plots between LC-MS/MS and HPLC-ECD. (A) Passing-Bablok regression and (B) Bland-Altman plot to compare VMA concentrations measured by LC-MS/MS and HPLC-ECD.

Abbreviations: LC-MS/MS, liquid chromatography-tandem mass spectrometry; HPLC-ECD, high–performance liquid chromatography coupled with electrochemical detection.

Supplemental Table 1. Summary of LC–MS/MS methods for quantification of HVA and VMA.

Reference	Column	Flow rate (µL/min)	Mobile phase (elution mode)	Internal standard	Run time	Sample volume (µl)	Injection volume (µl)	Ion mode	Sample preparation method	Mass spectrometer	Liquid chromatography
This study	C18 column (2.0 × 100 mm, 2.5 µm; Phenomenex)	300	0.1% (v/v) formic acid in water, 0.1% (v/v) formic acid in acetonitrile (gradient elution 15-90%)	HVA–d_5, VMA–d_3	6	50	10	NI	DS	Agilent 6460 TQ MS	Agilent 1260 HPLC

Clark, et al., 2017 [8]	Kinetex XB-C18 column (2.1 × 50 mm, 1.7 μm, Phenomenex)	400	0.05% (v/v) formic acid in water, 0.05% (v/v) formic acid in methanol (gradient elution with 0-95%)	VMA–d_3 HVA–$^{13}C_6{}^{18}O$	4	50	5	PI	DS	Waters Xevo TQ MS	Waters Acquity UPLC
Shen, et al., 2019 [9]	Synergi Fusion RP C18 column (2.0 ×100 mm, 3 μm, Phenomenex)	200	0.05% (v/v) formic acid in water, methanol (gradient elution with 40-100%)	HVA–d_3, VMA–d_3	3.5	50	5	NI	DS	AB Qtrap 4500 MS	Shimadzu LC-30AT
Grouzmann, et al., 2018 [a] [10]	Synergi Polar RP C18 column (2.0 mm × 50 mm, 2.5 μm, Phenomenex)	250	0.1% (v/v) formic acid in water, 0.1% (v/v) formic acid in methanol (gradient elution with 0-95%)	HVA–d_5, VMA–d_3	8	10	10	PI	DS	Waters Xevo TQ–S MS	Waters Acquity UPLC
Manini, et al., 2000 [11]	C16 amide column (50 × 4.6 mm, 5 μm, Supelco)	750	20mM formic acid in water, methanol (gradient elution with 18-80%)	NA	7	250	20	NI	DS	Perkin–Elmer Sciex API 365 TQ MS	Perkin–Elmer 200 binary system

Abbreviations: AB, applied biosystems; DS, dilute and shoot; HPLC, high–performance liquid chromatography; NA, not available; NI, negative ionization; PI, positive ionization; RP, reverse phase; TQ, triple quadrupole; UPLC, ultra–performance liquid chromatography.
